# Bioinoculant mediated regulation of signalling cascades in various stress responses in plants

**DOI:** 10.1016/j.heliyon.2023.e12953

**Published:** 2023-01-13

**Authors:** Disha Dasgupta, Anamika Paul, Krishnendu Acharya, Tatiana Minkina, Saglara Mandzhieva, Andrey Vladimirovich Gorovtsov, Nilanjan Chakraborty, Chetan Keswani

**Affiliations:** a Department of Botany, Scottish Church College, Kolkata 700006, India; b Molecular and Applied Mycology and Plant Pathology Laboratory, Department of Botany, University of Calcutta, Kolkata 700019, India; c Academy of Biology and Biotechnology, Southern Federal University, Rostov-on-Don 344090, Russia

**Keywords:** Abiotic stress, ACC deaminase, Microbiota, Rhizosphere, Siderophore, Solubilization

## Abstract

Bio-inoculation involves the association of plant with some beneficial microorganisms, and among these microbiotas, those bacteria which can promote plant growth and development are known as Plant Growth Promoting Rhizobacteria (PGPR). It can help a plant directly or indirectly, which includes root development, biological nitrogen (N_2_) fixation, stress tolerance, cell division and elongation, solubilization of Zinc, Phosphate, Potassium, soil health improvement and many more. PGPR have gained attention as it can be used as biofertilizers and helpful in bioremediation techniques, which in turn can reduce the chemical dependency in agriculture. PGPR mediated plant growth and stress management is developed by the virtue of the interaction of plant and microbial signalling pathways. On the other hand, environmental stresses are something to which a plant is always exposed irrespective of other factors. The present review is all about the better understanding of the convergence strategies of these signalling molecules and the ambiguities of signalling activities occurring in the host due to the interaction with PGPR under environmental stressed conditions.

## 1 Introduction

The rapid increase in global population has led to the high demand for food, and to fulfil the needs rate of production should be in equal proportion. Plant growth and yield are majorly dependent on soil health and the amendments present in it. For sustainable development in agriculture and by keeping the above scenario in mind, huge dependency on chemicals is being observed in recent days, which obviously has shown notable results in higher beneficiary products, but these results come with a huge cost. Using chemical fertilizers on a large scale can have a long-term effect on human health moreover it can reduce soil fertility permanently by affecting microbial diversity present in it [1]. Several successful attempts were done to reduce the chemical dependency on the agricultural field and replace these harmful products with some bioinoculants, which eventually will solve the same purpose and with no side effects. According to Ref. [2] microorganisms are popularly known for maintaining the biological and physiochemical equilibrium of soil.

Under the consequences of using chemical fertilizers to increase the fertility of soil or to maximize yield, the use of bioinoculants could be a sustainable mitigation option. Moreover, application of bioinoculant is safe for human and environmental health. Using bioinoculants in agricultural field is not a new practice rather it's an area where continuous innovations are being made to make a better version of it. Bioinoculants are a group of microorganisms that can potentially alter the soil condition for the betterment of plant growth and development. The course of interaction between microbiome and plant body is proven to be beneficial for both producers and purchasers. Soil-borne microorganisms can stimulate plant growth by promoting the availability of inaccessible nutrients in the soil [3]. Plants can take advantage of these microbes in the following ways: a) Phyto-stimulation (plant growth is promoted by the hormones released by microorganisms), b) PGPR (plant growth promoting rhizobacteria used as bioinoculant, c) Biological control agents (protect the plant body against abiotic stress) [4,5].

Microbial bioinoculats, particularly PGPR can directly enhance plant growth by producing bioactive substances and by increasing the nutrient bioavailability in the rhizosphere or indirectly facilitate plant development by eliminating other pathogenic effects as these can show antagonistic behaviour towards other phytopathogens [6]. Proximity of PGPR to the plant's roots (free-living or symbiotic association) not only enhance plant development rather evidence suggest their potential role against environmental stresses [[6], [7], [8]]. Multiple mineral solubilizing (Zinc, Phosphorus), hydrogen cyanide, and siderophore producing and N_2_ fixing strains are present in soil-borne microbiota which has a positive impact on the host plants [9,10]. Several reports suggested diverse bacterial genera as potent PGPR viz., *Achromobacter piechaudii, Azospirillum brasilense, Bacillus subtilis*, *Enterobacter asburiae, Enterobacter cloacae, Mycobacterium phlei, Pseudomonas syringae*, *Pseudomonas lini, Pseudomonas koreensis,* and many more [11,12], which have shown significant role on plant growth and on eliminating abiotic stresses.

The main objective of this review is to explore the field of bioinoculants, especially PGPR. The present work also investigates the interaction mechanism between plant body and microbiome which has led to sustainable agricultural production. This literature also promotes the use of bioinoculants over chemical compounds as chemical substances can have long-lasting side effects on mankind. The fact should be taken into consideration that bio-inoculation could grab the market of agribusiness without any harmful effects. In the following section the formulation techniques of PGPR, their features and most importantly the mechanism of action against different stress conditions are being elaborated. A special emphasis on the different stressed conditions against which application of PGPR as bioinoculants has already proven successful.

### 1.1 Growth promotion strategies of PGPR

Abundance of essential macro and micronutrients in rhizosphere region make them suitable for inhabitation of multiple microorganisms specifically for PGPR [13,14]. Plants can be benefited from the presence of PGPR as these microorganisms can promote plant growth and also help them to combat stressed conditions [15]. Initially PGPR was more often used for seed biotreatment to enhance the crop productivity and to promote natural growth, but soon the other useful effects were put forward viz. bioremediation, biofertilizer, phyto-stimulation and solubilization of different elements. All these applications of PGPR draw the attention of scientists to use them instead of chemical inoculation.

Based on the association of PGPR with the plant root, they are categorized into two groups i.e., extracellular PGPR (ePGPR) and intracellular PGPR (iPGPR). The former one supports the habitation of these bacteria in the rhizosphere zone or in the root cortical region (eg. *Azotobacter, Arthrobacter, Bacillus, Caulobacter Erwinia* and *Flavobacterium*) and the latter one supports rhizobacterial habitation in the intercellular space (eg: *Allorhizobium, Azorhizobium, Bradyrhizobium, Mesorhizobium* and *Rhizobium*) [16,17].

**Fig. 1:**
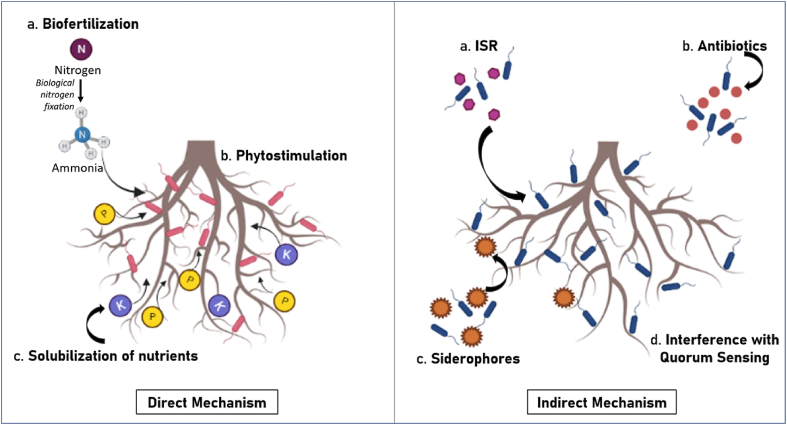
Direct and indirect mechanisms of plant growth from PGPR-host interactions.

Mechanism of plant growth promotion by PGPR can be broadly classified into direct and indirect method (Fig. 1). Various plant growth promoting activities facilitated by PGPR includes nitrogen fixation, inhibition of pathogenic interactions, phytohormone production, and many more [18]. Direct enhancement of plant growth incorporates biological fixation of N_2_ [19,20], solubilization of phosphorus [16], ACC Deaminase production [21], synthesis of Phytohormones [22], production of siderophore [23].

Indirect mechanism of plant growth enhancement by PGPR mainly includes biological control and plays an important role in plant defence system. Rhizobacteria can exclude the threat of phytopathogens like fungus, viruses, and bacteria by competing with them for food, exclusion of niche, synthesis of antifungal metabolites and induced systemic resistance (ISR). Antifungal substances like phenazines, 2, 4-diacetylphloroglucinol, tensin, pyoluteorin and viscosinamide are produced by rhizobacteria which makes them potent stress reliever [16]. ISR pathway can be activated by lipopolysaccharides (LPS), cyclic lipopeptides, siderophores, homoserine lactones, flagella and by certain other volatiles, these in turn produce jasmonate and ethylene signalling pathways to protect the host plants against different stresses [18]. Other beneficial effects of PGPR includes its utilisation as biofertilizers, for rhizoremediation, environmental stress eliminator and many more [19,20].

Another most prominent indirect mechanism includes antibiotic production, which are toxins with low molecular weight and have the potential to cease the growth of other microorganisms [24,25]. Rhizobacteria are the most potent antibiotic producers. *Bacillus subtilis*, *B*. *amyloliquefaciens* are reported to a variety of antifungal and antibacterial antibiotics, like bacillisin, subtilin, etc [26].

### 1.2 Convergence of signalling molecules

PGPR can synthesise lipo-chitooligosaccharides (LCOs) or nodulation factors (Nod factors) as signalling molecules which facilitates the symbiotic interaction between plant root and rhizobacteria and can promote nodule formation too [27]. They can also direct impact on lateral root formation specially when the host plants are exposed to stressed conditions. LCO shows a vital role in lateral root formation via auxin homeostasis (Buendia et al., 2018), which also indicates the presence of a proximity between plant and microbial signalling pathways. Plant receptors can recognize LCOs produced by bacterial associations like lysin motif receptor-like kinase (LysM-RLKs), containing oligosaccharide-binding LysM site. The interaction between LCOs with LysM activates signalling pathways which leads to nodulation. In case of leguminous plants, Nod factor receptors are present and can easily recognize the LCOs/Nod factors (NFs). Two such recently introduced NFRs (Nod factor receptors) are NFR1 and NFR5 [28]. Sometimes these pathways can be more active by the combination of LCOs and chitiologosaccarides (Cos). Interaction between chitiologosaccarides (Cos) and LYK/CERK1 receptors can activate plant defence mechanism by the activation of calcium (Ca^2+^) influx across the plasma membrane, synthesise Reactive oxygen species (ROS) and by activating MAPK (Mitogen-activated protein kinase) pathway. On the other hand, formation of ROS can be suppressed by LCOs in presence of phytopathogens.

**Fig. 2:**
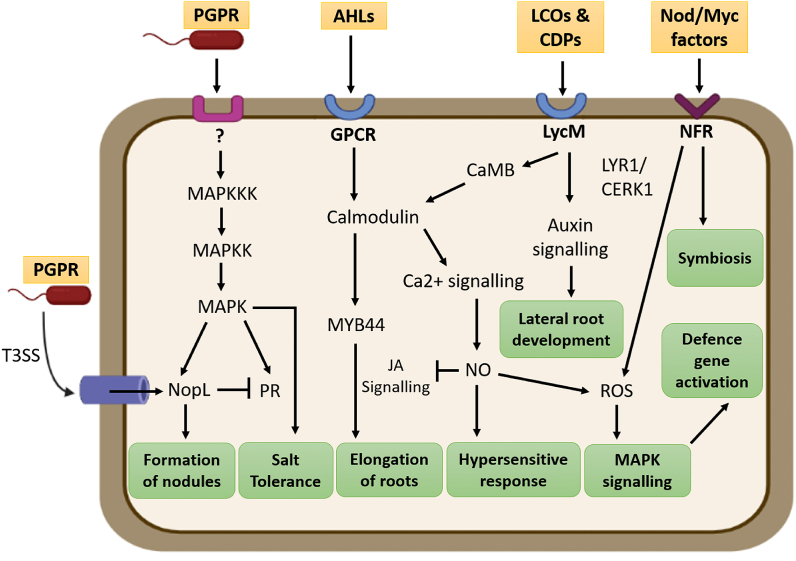
Probable interacting signalling pathways between rhizobacteria and the host plant body, which ultimately leads to several beneficial physiological pathways.

Rhizobial strains are also reported to produce an effector protein called NopL (Nodulation outer protein L) which can stimulate the formation of nodules in symbiotic association, this mechanism can be compared to that of T3SS (T3 effector proteins) produced by pathogenic microorganisms for invasion. Interaction between NopL and MAPK signalling can repress the early senescence of nodules and can also suppress the transcription of PR proteins like chitinase and glucanase. Ser/Thr kinase inhibitors and MAPKK inhibitors can block the phosphorylation of NopL, which suggests NopL to be a substrate of MAPK [29]. All these evidence show the significance of MAPK pathway as an interconnecting point to transmit signals in symbiotic association. Several other studies support the induction of calmodulin binding genes in presence of LCOs in plant. Calmodulin binding proteins (CaMB) are highly sensitive to calcium (Ca^2+^) and thus can control a large spectrum of target proteins [30]. Convergence of LCO with CaMB has potential role in calcium signalling pathway in host plant. Root elongation can also be promoted by MYB44, which is formed by the convergence of Acyl-homoserine lactones (AHLs) with G-protein coupled receptor (GPCR) [31]. All the afore-mentioned evidences suggest a stable convergence between PGPR and plant signalling pathways and as a result it can play a key role in maintaining plant health and improving defence system against phytopathogenic and environmental stresses. Fig. 2 represents the interface between rhizobial and plant signalling pathways.

Multiple studies reported Nitric oxide (NO) as an interlinking medium between PGPR and host plant signalling. Many rhizobacteria possess bacterial nitric oxide synthase (bNOS) which can oxidise l-arginine to l-citrulline, in presence of oxygen and resulting in the formation of NO [32]. Alternative pathway of Nitric oxide synthesis includes anaerobic denitrification by free living bacteria and in this interface nitrate (NO^−3^) is reduced to form nitrogen with the help of nitrate reductase (NR), nitrite reductase (NiR), NO reductase (NoR), and N_2_O reductase [33]. Denitrification can also occur in aerobic condition which involves periplasmic nitrate reductase (Nap) instead of membrane bound respiratory nitrate reductase (Nar). Another alternative pathway of bacterial NO synthesis involves heterotrophic nitrification which leads to oxidation of ammonia to hydroxylamine (NH_2_OH), NO^−2^, and NO^−3^. NO is a lipophilic diffusible bioactive compound which can take part in several signalling pathways including stress mediated and developmental pathways [32]. According to Ref. [32] NO plays a significant role in plant root development, on the other hand application of NO donors like nitrosoglutathione and SNP (sodium nitroprusside) can stimulate root growth in many plants, indicating the interaction between NO with IAA induced signal pathway [34]. Microscopic annotations verified similar anatomical adaptation in IAA and NO induced roots [32]. NO is also popular for participating plant developmental pathways through the interaction with ROS (Reactive oxygen species), phytohormone, post translational modification of a variety of proteins and Ca^2+^ regulated pathways. NO can actively regulate the production of ROS under various stressed condition [35,36].

## 2 Mechanism of PGPR mediated stress management

Plants are always exposed to different environmental stresses like salinity, heavy metal, water, and many more and to cope up with these adverse conditions the most common adaptations they show is the alteration in root morphology. Phytohormones like auxin, ethylene is reported to play a key role in root morphology modification. Indole acetic acid (IAA), which is produced in the arial parts of the plant body (mainly shoot) is transported to the lower part to promote cellular elongation and is specifically responsible for root growth [11]. Reports also suggest that lower concentration of auxin can promote root development and stimulate lateral root hairs formation, in contrast the previous statement higher concentration of the same phytohormone can suppress the root development. As per reports, the mechanism of root growth inhibition is somewhere modulated by the production of ethylene, which can be stated as ethylene and auxin is functionally antagonistic in nature. On the other hand, multiple other endophytic rhizobacteria like *Bacillus cereus, Bacillus megaterium, Klebsiella pneumoniae* and *Proteus mirabilis* are reported as potent ABA (Abscisic acid) producers, which is popularly known as stress hormone as it can also play a vital role in stress tolerance [37].

**Fig. 3:**
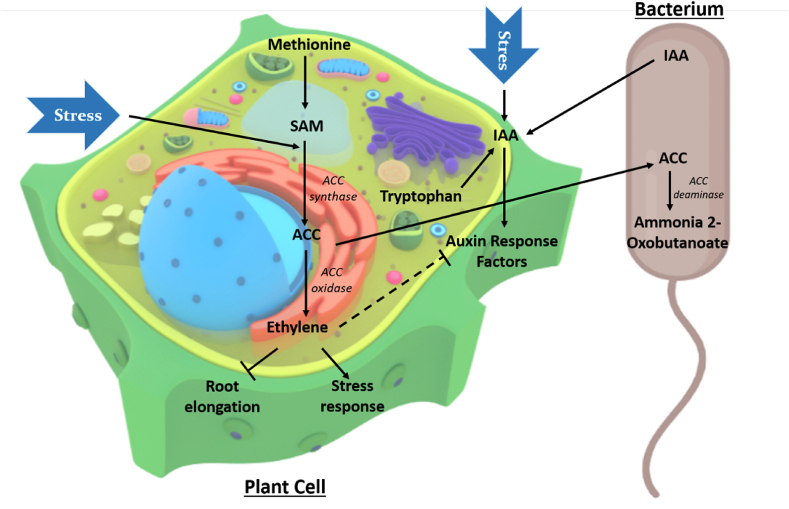
Mechanism of action of plant growth promoting rhizobacteria against stress factors via formation of ACC Deaminase which directly regulates the production of ethylene and can suppress root elongation (*SAM = S-Adenosyl Methionine).

Unsurprisingly PGPR associated plants can show similar root growth pattern, as these bacterial strains potentially produce IAA at an optimum level, which eventually leads to root growth and development of lateral root hairs [38,39]. Root growth promotion involves increased surface area which supports higher water and nutritional absorbance and is beneficial for the plant body [11]. PGPR can synthesise 1-aminocyclopropane-1-carboxylate (ACC) deaminase which can regulate plant growth under stressed condition [18]. Interestingly, these endophytic rhizobacteria with the help of ACC deaminase can hydrolyse ACC (1-aminocyclopropane-1-carboxylic acid) to form ammonia and utilize it as a source of nitrogen, on the other hand ACC is the precursor molecule for ethylene production. As a result of ACC hydrolysis ethylene production gets reduced accompanied with low auxin (IAA) level and this condition leads to the enhancement on root [40,41]. Several research works suggest ethylene as a stress modulator just because it plays a significant role in stress related signalling pathways. Molecular studies support that in abiotic stressed conditions, altered gene expression could be observed for ethylene production for those plants, which are previously treated with beneficial rhizobacteria [42]. Though, reduced level of ethylene can alter plant stress condition, but it is not the only one, proline is also known for its stress modulating activity. In fact, in case of proline mediated stress control pathways, plants which are treated with these endophytic rhizobacteria (*Arthrobacter, Burkholderia* and *Bacillus*) show a sharp increase in proline level [42]. *Azospirillum*, another popular root invading bacteria can also produce nitric oxide which can alter the morphology of root by reducing ethylene level under host's stressed condition. Though the exact mechanism of action of PGPR in stress tolerance is yet to be understood but several evidence support their potential against abiotic stressed conditions. A schematic diagram depicts the working principle of PGPR against environmental stress (Fig. 3).

**Fig. 4:**
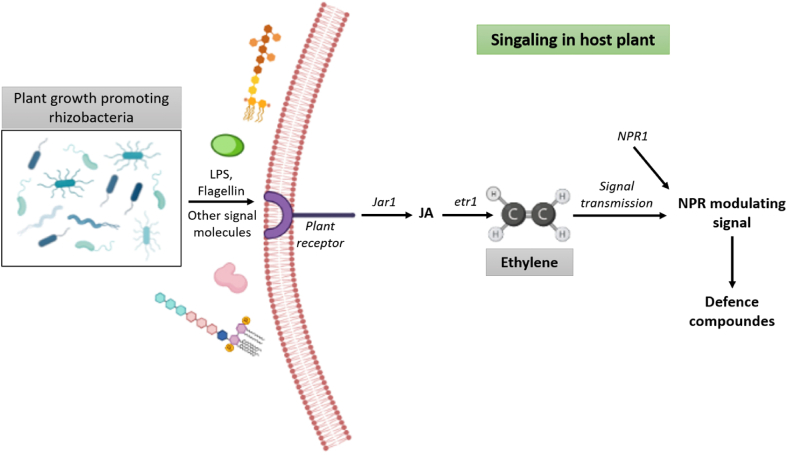
Defence mechanism promoted by PGPR against environmental stress.

In a broad-spectrum environmental stress in plant body is regulated by several enzymes like phenylalanine ammonia lyase (PAL), polyphenol oxidase (PPO), peroxidase (PO), superoxide dismutase (SOD), ascorbate peroxidase (APX), lipoxygenase (LOX), catalase (CAT) and proteinase inhibitors and all these enzymes are reported to be associated with induced acquired resistance (ISR) [43]. Such enzymes can also stimulate the production of phenolic compounds and phytoalexins which in turn will provide initial resistance against pathogenic interactions. Plant receptors (PRs) can sense the signal molecules like Volatile Organic Compounds (VOCs), Flagellin, LCOs, Cyclodipeptides (CDPs) and Lipopolysaccharides (LPS) which are produced by the rhizobacteria. Due to the previous interaction ethylene response mutant (ethylene *receptor1/ER1) and* JA response mutant (jasmonic acid receptor1/JAR1) become highly sensitive to ethylene and jasmonic acid respectively and lead to the formation of multiple defence molecules. Fig. 4 represents the formation of defensive substances via PGPR.

Systemic acquired resistance (SAR) is regulated by the endogenous accumulation of SA (salicylic acid) and its related signalling pathway. Pathogen related proteins (PR proteins) also play a vital role in SAR development. Though ISR and SAR pathways distinctly separated from each other by separate regulatory substances but both pathways show diverge downstream, in the presence of a regulatory mutant called, non-expressor pathogen related genes 1 (NPR1) [43]. NPR1 thus can regulate SA dependent expression of pathogen related proteins in SAR pathway and in JA and ethylene mediated ISR pathway.

### 2.1 Responses of bioinoculants against abiotic stresses

Plants are always prone to different stress factors, so it follows defensive mechanism to combat with the environmental stresses [44]. PGPR are the microbes which are used to stay alive in the rhizopheric regions of the Earth. By means of giving several supportive activities for plants either directly or indirectly those influence the growth and survival of the plants [45]. It induces the release and accumulation of plant growth regulating hormones like salicylic acid (SA), abscisic acid (ABA). These hormones play a centralized role by alteration of stomatal movement, transpiration, osmoregulation (by ABA); protection against pathogenic organisms (by SA) [46]. Ameliorative role played by PGPR against heat stress, water stress, salt stress, oxidative stress, cold stress, drought stress (abiotic stress) is already established by many workers [44,45,47]. PGPR controls multiple stress responses by the induction of ISR, pathogenesis related (PR) protein coding genes etc. [48]; increased in antioxidative enzyme activities, reduction in reactive oxygen species (ROS) generation [44], increased 1-aminocyclopropane-1-carboxylic acid (ACC) deaminase activity, reduced ethylene synthesis pathway etc. [49] during alleviation of abiotic stress. It plays an important role to save the plant from different abiotic stresses by altering their cellular activities and gene expression, some of which are discussed in Table 1.

**Table 1:** Effects of plant growth promoting rhizobacteria (PGPR) on alteration in plant gene expression to abiotic stresses.

Genes	Name of plant	Stress	PGPR applied	Alteration in expression	Reference
*COX1*	*Oryza sativa* L.	Drought stress	*Bacillus megaterium*, *Pseudomonas azotoformans*, *Rhizobium* sp.	Decreased	[50]
*AP2-EREBP*	*Oryza sativa* L.	Drought stress	*Rhizobium* sp.	Increased	[50]
*AtNRT1.2*	*Arabidopsis thaliana* (L.) Heynh.	–	Mixture of *Bacillus pumilus, Bacillus amyloliquefaciens, Bacillus safensis* and *Lysinibacillus xylanilyticus*	Increased	[51]
*GRAM*	*Oryza sativa* L.	Drought stress	*Pseudomonas azotoformans*	Increased	[50]
*NRAMP6*	*Oryza sativa* L.	Drought stress	*Pseudomonas azotoformans*, *Rhizobium* sp.	Increased	[50]
*NAM*	*Oryza sativa* L.	Drought stress	*Bacillus megaterium*, *Pseudomonas azotoformans*, *Rhizobium* sp.	Increased	[50]
*AtNRT1.1*	*Arabidopsis thaliana* (L.) Heynh.	–	Mixture of *Bacillus pumilus, Bacillus subtilis, Bacillus safensis* and *Lysinibacillus xylanilyticus*	Increased	[51]
*GST*	*Oryza sativa* L.	Drought stress	*Pseudomonas azotoformans*, *Rhizobium* sp.	Increased	[50]
*DHN*	*Oryza sativa* L.	Drought stress	*Bacillus megaterium*, *Pseudomonas azotoformans*, *Rhizobium* sp.	Increased	[50]
*EXP1*	*Oryza sativa* L.	Drought stress	*Bacillus megaterium*, *Rhizobium* sp.	Increased	[50]
*AtAMT1.1*	*Arabidopsis thaliana* (L.) Heynh.	–	Mixture of *Bacillus pumilus, Bacillus subtilis, Bacillus safensis* and *Lysinibacillus xylanilyticus*	Increased	[51]
*EXP2*	*Oryza sativa* L.	Drought stress	*Bacillus megaterium*, *Rhizobium* sp.	Increased	[50]
*EXP3*	*Oryza sativa* L.	Drought stress	*Bacillus megaterium*, *Rhizobium* sp.	Increased	[50]

### 2.2 Responses against heat stress

The major stress faced by the plants is due to the rise of global temperature i.e., called heat stress. It hampered the plant growth by altering photosynthetic rate, flowering, fruiting, influence root elongation, nitrogen mineralization, prompt N uptake, decreased nitrification etc. [44,52]. It accumulates amino acids and proline, boost up antioxidative enzyme activities like CAT, POD and SOD, shows scavenging activity against ROS [44]. Some thermotolerant property of PGPR has been studied to improve heat stress tolerance in plants. Moreover, it is clearly established that bacteria could be able to survive up to 60 °C temperature [49]. The result by Ref. [53] is already showed that *Bacillus cereus* SA1 strain could be used easily as a thermotolerant bacterium. They treated soybean plants with this strain, and it find out that SA1 strain ameliorate the effects of heat stress, like it reduces ABA and increases SA and amino acid content which are absolutely opposite to the heat stress. Simultaneously, it increases superoxide dismutase, ascorbic acid peroxidase, glutathione contents and potassium gradient. Inoculation of SA1 strain regulates the heat shock protein (HSP) expression, which showed overexpression of *GmLAX3* and *GmAKT2*. Overexpression of these two genes reduces the production of ROS. PGPR can ameliorate heat stress response by increasing ACC deaminase production in maize, wheat and other grain crops [49]. *Bacillus cereus* induces heat tolerance by producing ACC-deaminase (0.76–0.9 μM/mg protein/h) in tomato (*Solanum lycopersicum* L.) plants. It also increases the exopolysaccharide synthesis (0.66–0.91 mg/mL) that influence on growth in tomato plant [54]. Seed treatment of the wheat cultivars Sids1 and Olivin with *Azospirillum brasilense* NO40 and *Bacillus amyloliquefaciens* UCMB5113 showed improved heat tolerance in Olivin in comparison with Sids1. Biochemical analysis showed reduction in enzymes activities of ascorbate-glutathione redox cycle, reduces ROS generation and cell damage, and activates heat shock proteins [55]. *Bacillus aryabhattai* strain SRB02 treated soybean plants showed increased root and shoot development, constant ABA production, ABA-mediated stomatal closure, increases the level of indole acetic acid (IAA), jasmonic acid (JA), gibberelic acids like GA4, GA7 and GA12. SRB02 strain simultaneously proved it's used in biofertilizer and in other soil amendments [56]. Misra et al. [57] showed many species of *Bacillus* increases ACC deaminase production in many plants against heat stress response. Seeds of wheat cultivar (HUW-234) treated with *Pseudomonas aeruginosa* (strain 2CpS1) in elevated temperature showed reduction in membrane damage and gradual increased in ACC deaminase activity [49]. Sorghum seedling inoculated with *Psuedomonas aeruginosa* AKM-P6 strain improved the production of proline, amino acids, sugars, chlorophyll, as well as helped the plant to survive up to 47–50 °C temperature [58]. The effects of PGPR to alleviate heat stress are presented in Table 2.

**Table 2:** Effects of plant growth promoting rhizobacteria (PGPR) on plants to abiotic stresses.

Plant stress	Target plant	PGPR	Effects on plant	Reference
Heat stress	Tomato (*Solanum lycopersicum* L.)	*Bacillus cereus*	Increased production of exo- polysaccharide, cleavage of ACC- deaminase	[54]
	Wheat (*Triticum aestivum* L.)	*Bacillus amyloliquefaciens, Azospirillum brasilense*	ROS reduction, reduced cell damage, pre-activation of heat shock proteins, enzymes of the ascorbate-glutathione redox cycle reduce their activity	[55]
	Soybean (*Glycine* max (L.) Merr.)	*Bacillus aryabhatthai*	ABA production	[56]
	Many plants	*Bacillus* spp.	Enhances ACC deaminase production	[57]
	Wheat (*Triticum aestivum* L.*)*	*Pseudomonas aeruginosa*	Increase ACC deaminase activity, reduce membrane damage	[49]
	Sorghum (*Sorghum bicolour* (L.) Moench)	*Psuedomonas aeruginosa*	Increases proline, amino acids, sugars, chlorophyll content	[58]
Drought stress	Wheat (*Triticum aestivum* L.*)*	*Bacillus amyloliquefaciens*, *Agrobacterium fabrum*	Increases biomass content and grain yield	[59]
	Maize (*Zea mays* L.)	*Cupriavidus necator, Pseudomonas fluorescens*	Increases shoot biomass	[60]
	Sweet corn (*Zea mays* L.)	*Pseudomonas fluorescens*	Increases ear and canned seed yield of 44% and 27%	[61]
	Foxtail millet (*Setaria italic* L.)	*Pseudomonas fluorescens*, *Enterobacter hormaechei*, *Pseudomonas migulae*	Produce EPS, higher ACC deaminase activity	[62]
	Velvet bean (*Mucuna pruriens* (L.) DC.)	*Bacillus* spp., *Enterobacter* spp.	Produces ACC-deaminase and IAA, reduces plant ACC and root ethylene level	[58]
	Chickpea (*Cicer arietium* L.)	*Pseudomonas putida*	Accumulates osmolyte, increases the expressions of stress-responsive gene, scavenge ROS	[63]
	Wheat (*Triticum aestivum* L.)	*Variovorax paradoxus, Pseudomonas* spp. *Achromobacter* spp., *Ochrobactrum anthropi*	Promote N_2_ fixation and produces ACC-deaminase	[64]
	Maize (*Zea mays* L.)	*Streptomyces pseudovenezuelae*, *Arthrobacter* *arilaitensis*	Increase in plant growth	[11]
	Lettuce (*Lactuca sativa* L.)	*Azospirillum* sp.	Enhances ascorbic acid, chlorophyll content, antioxidant capacity increased	[65]
	Maize (*Zea mays* L.)	*Herbaspirillum seropedicae*, *Azospirillum brasilense*	Increases biomass, reduces ethylene and ABA level	[66]
	Jujube (*Ziziphus jujuba* Mill.)	*Pseudomonas lini, Serratia plymuthica*	Increases in plant height, root and shoot dry weight, water content, antioxidant enzyme activities and decreases ABA level	[67]
	White clover (*Trifolium repens* L.)	*Bacillus megaterium, Pseudomonas putida*	Increases water and plant nutrient contents, reduces stress enzyme activities and stomatal conductance	[68]
	Maize (*Zea mays* L.)	*Pseudomonas pseudoalcaligenes*	Increases proline content, root and shoot weight	[69]
	Maize (*Zea mays* L.)	*Planomicrobium chinense*, *Bacillus cereus*	Decreases antioxidant enzyme activity (catalase, peroxidase), increases root and shoot dry weight	[70]
Salinity stress	French bean (*Phaseolus vulgaris* L.)	*Aneurinibacillus aneurinilyticus*, *Paenibacillus* sp.	Increases production of hydrogen cyanide, IAA and siderophore, facilitated ACC-deaminase activity	[71]
	Wheat (*Triticum aestivum* L.)	*Stenostrophomonas maltophilia*	Increases K^+^ uptake, growth, yield and antioxidant enzyme activity	[48]
	Lettuce (*Lactuca sativa* L.)	*Azospirillum* sp.	Enhances ascorbic acid, chlorophyll content, antioxidant capacity increased	[65]
	Chickpea (*Cicer arietinum* L.)	*Bacillus subtilis* and *Mesorhizobium ciceri*	Proline accumulation increases	[72]
	Rice (*Oryza sativa* L.)	*Enterobacter* sp.	Reduction in ethylene production and antioxidative enzymatic activities, enhances seedling growth	[73]
	Oats (*Avena sativa* L.)	*Klebsiella* sp.	Increases dry root and shoot weight, growth, water content	[74]
	Tall fescue (*Festuca arundinacea* Schreb.)	*Enterobacter ludwigii*	Produces plant hormone, phosphate solubilization, increases nitrogen fixation, growth, tolerance, and productivity of the plant	[75]
	Chili (*Capsicum annuum* L.)	*Bacillus* spp., *Alcaligenes* spp., *Proteus* spp., *Aneurinibacillus aneurinilyticus*	Increases shoot and root length	[76]
	Rice (*Oryza sativa* L.)	*Bacillus amyloliquefaciens*	Modulates gene expression	[77]
	Rice (*Oryza sativa* L.)	*Bacillus* sp.	Increases the growth and development by increasing biomass content of seedling by enhancing the IAA production and augmentation of deaminase enzyme activity	[57]
	Rice (*Oryza sativa* L.)	*Thalassobacillus denorans*, *Oceanobacillus kapialis*	Increases seed germination, root and shoot growth, protein, chlorophyll and nutrient contents, reduction in accumulation of sodium ion	[78]
	Canola (*Brassica napus* L.)	*Enterobacter cloacae*	Enhanced root, shoot, and chlorophyll contents, hormonal content and reduces malondialdehyde and ethylene content	[79]
	Pea (*Pisum sativum* L.)	*Acinetobacter bereziniae, Enterobacter ludwigii, Alcaligenes faecalis*	Altered in antioxidative enzyme activity, proline, chlorophyll, and soluble sugar content	[80]
Oxidative stress	Maize (*Zea mays* L.)	*Herbaspirillum seropedicae* and *Azospirillum brasilense*	Increases biomass, reduces ethylene and ABA level	[66]
Alkalinity stress	Maize (*Zea mays* L.)	*Bacillus* sp., *Alcaligenes* sp.	Increases soluble sugar and photosynthetic pigment content, enhanced in dehydrogenase, betaglucosidase and alkaline phosphatise activity	[81]
	Wheat (*Triticum aestivum* L.)	*Bacillus clausii*	Increases root, shoot growth and grain yield	[82]
	Wheat (*Triticum aestivum* L.)	*Virgibacillus marismortui*	Increases root, shoot growth and grain yield	[82]
	Wheat (*Triticum aestivum* L.)	*Lysinibacillus* sp.	Increases germination of seeds and vegetative growth	[83]
	Wheat (*Triticum aestivum* L.)	*Bacillus simplex*	Increases plant growth, decrease in pH of rhizospheric soil, increased in root P content	[84]
	Faba beans (*Vicia faba* L.)	*Bacillus subtilis*	Increases seed germination, plant growth and yield	[85]

### 2.3 Responses against drought stress

The changes of temperature are conjoined with the demand of water for progress of physical activities for a plant. Rainfall is the only source of water for the farmers, but the reduced level of rainfall due to environmental changes or manmade activities is a matter of concern for the people. Because it lower down the level of ground water, and cause hamper to plant growth and development. It resulted to drought [45]. Due to the drought stress, plant responses toward the higher production of ethylene, influence ACC oxidase that prompts ACC exudation. This ACC is the precursor of ethylene biosynthesis pathway. Whereas PGPR played an important role in the degradation of ACC due to the activity of ACC deaminase. It degrades ACC into ammonia and α-ketobutyrate, so that is controls ethylene production and helps the plant in normal growth and development [44]. Inoculation of several bacterial strains in the plants showed their effects towards the completion of plant's normal cycle by production of phytohormones and volatile organic compounds, increases signalling pathways, regulation of antioxidant enzymes activity, production of exopolysaccharides (EPS) that improves water potential and soil aggregation [15]. EPS form protective rhizosheath for decreasing waterlessness time around soil aggregates. It helps to hold more water for the plant to make it safe from the drying [70]. *Cupriavidus necator* strain 1C2 and *Pseudomonas fluorescens* strain S3X inoculation increases shoot biomass up to 89% in maize. These two bacteria are already known as IAA producing PGPR [60]. Co-application of ACC deaminase producing PGPR i.e., *Bacillus amyloliquefaciens* with timber waste biochar increases productivity under drought stress condition viz., about 59% in grain weight, 118% in photosynthetic rate, 114% in chlorophyll *a*, 123% in chlorophyll *b* content, 73% of transpiration rate [59]. *Herbaspirillum seropedicae* Z-152 and *Azospirillum brasilense* SP-7 strains shows negative effect on drought stress by increasing 29.5% and 26% biomass content in inoculated maize plants. It also lowers ethylene and ABA levels [66]. Ortiz et al. [68] stated that inoculation of *Bacillus megaterium*, *Pseudomonas putida* helped white clover plant to survive in drought stress by increasing nutrient and water content in plant, reduces stress enzyme activities and stomatal conductance. *Streptomyces pseudovenezuelae* and *Arthrobacter arilaitensis* strains inoculated in *Zea mays* show significant increase in physiological parameters to combat with the drought stress [86]. ACC deaminase activity carrying four strains of *Pseudomonas fluorescens* (P1, P3, P8, and P14) together used as a combination treatment. These helped to increase in ear and canned seed yield of 44% and 27% in sweet corn, respectively [61]. Niu et al. [62] isolated different strains of bacteria, namely *Pseudomonas fluorescens, Enterobacter hormaechei,* and *Pseudomonas migulae* from foxtail millet which is a drought resistant plant. These strains augment seed germination and growth of young seedling, enhances EPS producing activity and ACC deaminase activity. *Pseudomonas lini* and *Serratia plymuthica* found to be increased in jujube plant height, root and shoot dry weight, water content, antioxidant enzyme activities and decreases ABA level [67]. Table 2 shows a list of PGPR with their effects on target plants. Yasmin et al. [69] showed that *Pseudomonas pseudoalcaligenes* increases proline content, root and shoot weight in maize plant during drought stress. This study proves that alteration of enzymes activities, gene expression and physiological activities can protect the plant against drought.

### 2.4 Responses against salinity stress

Salinity is referred to as the deleterious effects of sodium chloride and other salt compounds in the environment. It causes harmful effects on plants particularly on growth and development by altering photosynthetic rate, stomatal closure, etc. Like drought stress, salinity stress also induces the production of ethylene in plant body. So, it requires ACC deaminase to reduce the ACC (precursor of ethylene synthesis) content in the plant. It cleaves ACC into ammonia and α-ketobutyrate to reduce the ethylene formation and ultimately normalize the plant growth [44]. PGPR like *Bacillus amyloliquefaciens* NBRISN13 strain modulates gene expression in rice plant [77], *Stenostrophomonas maltophilia* increases K^+^ uptake, enhances growth and yield, induced antioxidant enzyme activity in *Triticum aestivum* during salt stress [48]. Chickpea plant shows an endophytic relationship when it was co-inoculation with *Mesorhizobium ciceri* IC53 strain and *Bacillus subtilis* NUU4 strain during salinity stress. It markedly showed a level of increase in proline accumulation which helped to sequester the hydrogen peroxide to provide strengthen by providing tolerance against salt. It also reduces the infection root rot caused by *Fusarium solani* [72]. Patel et al. [76] showed increased in root and shoot length in chilli plant when it was inoculated with *Bacillus* spp., *Alcaligenes* spp., *Proteus* spp., *Aneurinibacillus aneurinilyticus* during salt stress than the non-inoculated control plant. *Bacillus* sp. inoculated rice plant alleviate the effects of salt stress by enhancing the IAA production and augmentation of deaminase enzyme activity that leads toward the increase in growth and development of seedling by increasing biomass content [57]. *Klebsiella* sp. inoculated oats plant showed increased dry root and shoot weight, growth, water content as opposite response to salt stress [74]. Rice plant inoculated with *Thalassobacillus denorans* NCCP-58 strain and *Oceanobacillus kapialis* NCCP-76 strain showed reduction in accumulation of sodium ion, increased seed germination, root and shoot growth, protein, chlorophyll and nutrient contents [78]. Enhanced root, shoot, and chlorophyll contents were observed when canola plant inoculated with *Enterobacter cloacae* HSNJ4 strain. It also resulted in an increase in hormonal content and reduces malondialdehyde and ethylene content [79]. A study of [80] explained the role of *Acinetobacter bereziniae* IG 2 strain, *Enterobacter ludwigii* IG 10 strain, *Alcaligenes faecalis* IG 27 strain as PGPR in the alleviation of salt stress in pea (*Pisum satium*) plant. It was a field trial with 100 mM NaCl exposure to the pea plant. They reduce H_2_O_2_ content and lower down the electrolytic leakage. It altered several biochemical parameters like antioxidative enzyme activity, proline, chlorophyll, and soluble sugar content. This finding was concluded that *Alcaligenes faecalis* IG 27 strain is more potent to enhance the plant growth under salt stress condition [80] (Table 2).

### 2.5 Responses against oxidative stress

The climatic change is a natural process to us, but the climatic change globally causes a drastic change in the environmental factors that produce huge disturbance in biological or physiological activities of plants. Global climatic change mainly plays a role in the production of ROS. Oxidative stress develops when plants failed to produce sufficient concentration of antioxidants which helped the plant cells to neutralize reactive species (O^−^, OH^−^, and H_2_O_2_^−^). Increase in the generation of reactive species leads toward the damage of plant cellular components like proteins, lipids, nucleic acids and metabolites. Rhizobacteria along with plant synthesized osmolytes in plant cell are required for the induction of defence response against this stress to reduce ROS. To neutralize osmotic pressure, osmolytes (like amino acids, proline, methylamines, sugars, methylsulfonium compounds, polyols, and urea) can help the cells under stress conditions [44]. *Herbaspirillum seropedicae* Z-152 and *Azospirillum brasilense* SP-7 are two selected PGPR strain as shown in Table 2. Experiment on maize plant inoculated with these two strains showed increased proline accumulation i.e., 4- fold high in *H. seropedicae* inoculated plant as compared to the *A. brasilense* inoculated plant i.e., about 2-fold high than the non-inoculated control plant. So, it confers a good level of osmoregulation indication [66].

### 2.6 Responses against alkalinity stress

Alkalinity is another parameter for the soil i.e., designated as the soil rich in sodium salts like sodium carbonate (Na_2_CO_3_), sodium chloride (NaCl), and sodium hydrogen carbonate (NaHCO_3_) that increases soil pH. High pH plays an inhibitory role on non-alkaliphiles present in the rhizosphere. It influences on the soil chemistry, biology and the physiological activities, growth and development of the plant [45]. So, soil pH is directly linked with the crop productivity. Soils are categorized by The United States Department of Agricultural National Resources Conservation Service on the basis of their pH viz., ultra-acidic with pH < 3.5, extremely acidic with pH 3.5–4.4, very strongly acidic with pH 4.5–5.0, strongly acidic with pH 5.1–5.5, moderately acidic with pH 5.6–6.0, slightly acidic with pH 6.1–6.5, neutral with pH 6.6–7.3, slightly alkaline with pH 7.4–7.8, moderately alkaline with pH 7.9–8.4, strongly alkaline with pH 8.5–9.0, and very strongly alkaline with pH > 9.0 [87]. PGPR helps the plants to grow on the alkaline soil by moderating their physiological functions. Two strains of *Bacillus* sp. (NBRI YE4.4 and NBRI YE1.3), *Alcaligenes* sp. NBRI NB2.5 strain inoculated maize plant shows an elevation in seed germination process and biomass content under alkalinity stress. Inoculation of NBRI YE4.4 strain of *Bacillus* sp. is more accurate to perform their work by increasing soluble sugar and photosynthetic pigment content; enhanced in dehydrogenase, betaglucosidase and alkaline phosphatise activity. Therefore, this strain is used to alleviate the soil health and plant growth of saline soil [81]. *Bacillus clausii*, *Virgibacillus marismortui*, *Lysinibacillus* sp. and *Bacillus simplex* inoculated separately in wheat plant. *Bacillus clausii* and *Virgibacillus marismortui* show a level of increase in root, shoot growth and grain yield [82]. *Lysinibacillus* sp. shows increase in germination of seeds and vegetative growth [83], *Bacillus simplex* helped the plant under alkalinity stress by increasing plant growth, root P content, decrease in pH of rhizospheric soil [84] as shown in Table 2. Yousef [85] stated that *Bacillus subtilis* increases in seed germination, plant growth and yield in faba beans plant. This study provides the role of PGPR in plant growth under alkaline stress to ameliorate the negative effects of alkalinity.

## 3 Future prospects and challenges

The use of PGPR as soil stimulant that helps to improve soil health and give protection to the plants against harmful microbes, several environmental stresses. Whereas, PGPR showed its substantial role as an eco-friendly approach to manage stresses, by reducing the use of chemical fertilizers, other chemical agents that have side effects on the plant health PGPR also plays their role by producing several bioactive phytohormones like indole acetic acid (IAA), cytokinin and gibberellins [88], hydrogen cyanide (HCN), ammonia (NH_3_), altered the volatiles and content [89], some active enzymes against heavy metal, salinity, drought stress [90]. It improves the cycling of affecting elements and nutrients. It altered the activities of ACC deaminase, catalase, production of exopolysaccharides, phytohormones, organic acids, osmolytes etc. [89,91]. The other advantages are, it helps in rhizoremediation, growth enhancement, elicit the production of biomolecules, induction of systemic resistance against disease etc. [19,20]. Hence, this literature review suggests that the research in PGPR mediated induction of stress tolerance in plants by enhancing growth, rhizospheric competence, safety toward nature, mass production etc. will provide a validate eco-friendly, long term affective study. Though the market demand for food crops is rapidly increasing and so is the use of chemical fertilizers and pesticides for sustaining crop production. On the other hand, farmers have an eco-friendly and cost-effective alternative of using PGPR to increase the crop production. Whereas the challenges faced by the farmers is the lack of proper training on the applications and delivery methods of the commercial formulations [92]. In addition, the markets are flooded with spurious and low-quality products [93].

## 4 Conclusion

PGPR are the bacterial strains which used in the treatment of the plants to alleviate the stress responses. PGPR helps to increase ACC deaminase activity, increases osmolyte production, produce sufficient concentration of antioxidants, regulates heat shock protein (HSP), increases antioxidative enzyme activities like CAT, POD and SOD etc. However, current study shows the potentiality of PGPR against stresses by the practical references which are given throughout the manuscript to prove its ability. Though efficient future strategies for their selection, screening and characterization, biosafety assessment, formulation and delivery methods needs to be developed for harnessing their maximum potential.

## Author contribution statement

All authors listed have significantly contributed to the development and the writing of this article.

## Funding statement

Dr. Chetan Keswani gratefully acknowledges financial support from the project of the Ministry of Science and Higher Education of the Russian Federation on the Young Scientist Laboratory within the framework of the Interregional Scientific and Educational Center of the South of Russia (no. LabNOTs-21-01AB, FENW-2021-0014) and the Strategic Academic Leadership Program of the Southern Federal University (“Priority 2030”).

## Data availability statement

No data was used for the research described in the article.

## Declaration of interest's statement

.

## References

[1] Chaudhary T., Dixit M., Gera R., Shukla A.K., Prakash A., Gupta G., Shukla P. (2020). Techniques for improving formulations of bioinoculants. 3 Biotech.

[2] Alegria-Terrazas R., Giles C., Paterson E., Robertson-Albertyn S., Cesco S., Mimmo T., Pii Y., Bulgarelli D. (2016). Plant–microbiota interactions as a driver of the mineral turnover in the rhizosphere. Adv. Appl. Microbiol..

[3] Rawat N., Sharma M., Suyal D.C., Singh D.K., Joshi D., Singh P., Goel R. (2019). Psyhcrotolerant bio-inoculants and their co-inoculation to improve *Cicer arietinum* growth and soil nutrient status for sustainable mountain agriculture. J. Soil Sci. Plant Nutr..

[4] Tahir H.A.S., Gu Q., Wu H., Niu Y., Huo R., Gao X. (2017). *Bacillus* volatiles adversely affect the physiology and ultra-structure of *Ralstonia solanacearum* and induce systemic resistance in tobacco against bacterial wilt. Sci. Rep..

[5] Tang Y., Kang H., Qin Z., Zhang K., Zhong Y., Li H., Mo L. (2020). Significance of manganese resistant *Bacillus cereus* strain WSE01 as a bioinoculant for promotion of plant growth and manganese accumulation in *Myriophyllum verticillatum*. Sci. Total Environ..

[6] Singh M., Bhasin S., Madan N., Suyal D.C., Soni R., Singh D. (2021). Microbiological Activity for Soil and Plant Health Management.

[7] Keswani C., Dilnashin H., Birla H., Singh S.P. (2019). Unravelling efficient applications of agriculturally important microorganisms for alleviation of induced inter-cellular oxidative stress in crops. Acta Agric. Slov..

[8] Marastoni L., Pii Y., Maver M., Valentinuzzi F., Cesco S., Mimmo T. (2019). Role of *Azospirillum brasilense* in triggering different Fe chelate reductase enzymes in cucumber plants subjected to both nutrient deficiency and toxicity. Plant Physiol. Biochem..

[9] Lalitha S. (2017). Sustainable Agriculture towards Food Security.

[10] Patil H.J., Solanki M.K. (2016). Microbial Inoculants in Sustainable Agricultural Productivity.

[11] Dimkpa C., Weinand T., Asch F. (2009). Plant–rhizobacteria interactions alleviate abiotic stress conditions. Plant Cell Environ..

[12] Singh H.B., Keswani C., Reddy M.S., Royano E.S., García-Estrada C. (2019).

[13] Oleńska E., Małek W., Wójcik M., Swiecicka I., Thijs S., Vangronsveld J. (2020). Beneficial features of plant growth-promoting rhizobacteria for improving plant growth and health in challenging conditions: a methodical review. Sci. Total Environ..

[14] Tripathi R., Tewari R., Singh K.P., Keswani C., Minkina T., Srivastava A.K., De Corato U., Sansinenea E. (2022). Plant mineral nutrition and disease resistance: a significant linkage for sustainable crop protection. Front. Plant Sci..

[15] Notununu I., Moleleki L., Roopnarain A., Adeleke R. (2022). Effects of plant growth-promoting rhizobacteria on the molecular responses of maize under drought and heat stresses: a review. Pedosphere.

[16] Bhattacharyya P.N., Jha D.K. (2012). Plant growth-promoting rhizobacteria (PGPR): emergence in agriculture. World J. Microbiol. Biotechnol..

[17] Singh I. (2018). Plant growth promoting rhizobacteria (PGPR) and their various mechanisms for plant growth enhancement in stressful conditions: a review. Eur. J. Bot..

[18] Glick B.R. (2012).

[19] Joshi A.U., Andharia K.N., Patel P.A., Katadiya R.J., Kothari R.K., Singh Tomar A., Vijay B., Mandaliya V.B. (2019). Green Biotechnology.

[20] Joshi A.U., Andharia K.N., Patel P.A., Kotadiya R.J., Kothari R.K. (2019).

[21] Kang B.G., Kim W.T., Yun H.S., Chang S.C. (2010). Use of plant growth promoting rhizobacteria to control stress responses of plant roots. Plant Biotechnol. Rep..

[22] Ahemad M., Khan M.S. (2012). Ecological assessment of biotoxicity of pesticides towards plant growth promoting activities of pea (*Pisum sativum*)- specific *Rhizobium* sp. strain MRP1. Emir. J. Food Agric..

[23] Rajkumar M., Ae N., Prasad M.N.V., Freitas H. (2010). Potential of siderophoreproducing bacteria for improving heavy metal phytoextraction. Trends Biotechnol..

[24] Bakker P., Berendsen R.L., Doornbos R.F., Wintermans P.C.A., Pieterse C.M.J. (2013). The rhizosphere revisited: root microbiomics. Front. Plant Sci..

[25] Keswani C., Singh H.B., García-Estrada C., Caradus J., He Y.W., Mezaache-Aichour S., Glare T.R., Borriss R., Sansinenea E. (2020). Antimicrobial secondary metabolites from agriculturally important bacteria as next-generation pesticides. Appl. Microbiol. Biotechnol..

[26] Chang W.T., Chen Y.C., Jao C.L. (2007). Antifungal activity and enhancement of plant growth by Bacillus cereus grown on shellfish chitin wastes. Bioresour. Technol..

[27] Maillet F., Poinsot V., André O., Puech-Pagès V., Haouy A., Gueunier M., Cromer L., Giraudet D., Formey D., Niebel A., Martinez E.A., Dénarié J. (2011). Fungal lipochitooligosaccharide symbiotic signals in arbuscular mycorrhiza. Nature.

[28] Oldroyd G.E. (2013). Speak, friend, and enter: signalling systems that promote beneficial symbiotic associations in plants. Nat. Rev. Microbiol..

[29] Ge Y.Y., Xiang Q.W., Wagner C., Zhang D., Xie Z.P., Staehelin C. (2016). The type 3 effector NopL of *Sinorhizobium* sp. strain NGR234 is a mitogen-activated protein kinase substrate. J. Exp. Bot..

[30] Zeng H., Xu L., Singh A., Wang H., Du L., Poovaiah B.W. (2015). Involvement of calmodulin and calmodulin-like proteins in plant responses to abiotic stresses. Front. Plant Sci..

[31] Jalmi S.K., Sinha A.K. (2022). Ambiguities of PGPR-Induced plant signaling and stress management. Front. Microbiol..

[32] Grover M., Bodhankar S., Sharma A., Sharma P., Singh J., Nain L. (2021). PGPR mediated alterations in root traits: way toward sustainable crop production. Front. Sustain. Food Syst..

[33] Meilhoc E., Boscari A., Bruand C., Puppo A., Brouquisse R. (2011). Nitric oxide in legume–Rhizobium symbiosis. Plant Sci..

[34] Chakraborty N., Acharya K. (2017). NO way”! Says the plant to abiotic stress. Plant Gene.

[35] Chakraborty N. (2021). Salicylic acid and nitric oxide cross-talks to improve innate immunity and plant vigor in tomato against *Fusarium oxysporum* stress. Plant Cell Rep..

[36] Simontacchi M., Galatro A., Ramos-Artuso F., Santa-María G.E. (2015). Plant survival in a changing environment: the role of nitric oxide in plant responses to abiotic stress. Front. Plant Sci..

[37] Karadeniz A., Topcuoğlu Ş.F., Inan S. (2006). Auxin, gibberellin, cytokinin and abscisic acid production in some bacteria. World J. Microbiol. Biotechnol..

[38] Belimov A.A., Dodd I.C., Hontzeas N., Theobald J.C., Safronova V.I., Davies W.J. (2009). Rhizosphere bacteria containing 1-aminocyclopropane-1-carboxylate deaminase increase yield of plants grown in drying soil via both local and systemic hormone signalling. New Phytol..

[39] Keswani C., Singh S.P., Cueto L., García-Estrada C., Mezaache-Aichour S., Glare T.R., Borriss R., Singh S.P., Blázquez M.A., Sansinenea E. (2020). Auxins of microbial origin and their use in agriculture. Appl. Microbiol. Biotechnol..

[40] Long H.H., Schmidt D.D., Baldwin I.T. (2008). Native bacterial endophytes promote host growth in a species-specific manner; phytohormone manipulations do not result in common growth responses. PLoS One.

[41] Saleem M., Arshad M., Hussain S., Bhatti A.S. (2007). Perspective of plant growth promoting rhizobacteria (PGPR) containing ACC deaminase in stress agriculture. J. Ind. Microbiol. Biotechnol..

[42] Sziderics A.H., Rasche F., Trognitz F., Sessitsch A., Wilhelm E. (2007). Bacterial endophytes contribute to abiotic stress adaptation in pepper plants (*Capsicum annuum* L.). Can. J. Microbiol..

[43] Annapurna K., Kumar A., Kumar L.V., Govindasamy V., Bose P., Ramadoss D. (2013). Bacteria in Agrobiology: Disease Management.

[44] Younas R., Gul S., Ahmad R., Khan A.R., Khan M., Anwar T., Qureshi H. (2021).

[45] Enebe M.C., Babalola O.O. (2018). The influence of plant growth-promoting rhizobacteria in plant tolerance to abiotic stress: a survival strategy. Appl. Microbiol. Biotechnol..

[46] Bashir T., Naz S. (2020). Plant growth promoting rhizobacteria in combination with plant growth regulators attenuate the effect of drought stress. Pakistan J. Bot..

[47] Maitra S., Brestic M., Bhadra P., Shankar T., Praharaj S., Palai J.B., Shah M.M.R., Barek V., Ondrisik P., Skalický M., Hossain A. (2022). Bioinoculants—natural biological Resources for sustainable plant production. Microorganisms.

[48] Singh R.P., Jha P.N. (2017). The PGPR *Stenotrophomonas maltophilia* SBP-9 augments resistance against biotic and abiotic stress in wheat plants. Front. Microbiol..

[49] Meena H., Ahmed M.A., Prakash P. (2015). Amelioration of heat stress in wheat, *Triticum aestivum* by PGPR (*Pseudomonas aeruginosa* strain 2CpS1). Biosci. Biotechnol. Res..

[50] Omar S.A., Fetyan N.A.H., Eldenary M.E., Abdelfattah M.H., Abd-Elhalim H.M., Wrobel J., Kalaji H.M. (2021). Alteration in expression level of some growth and stress-related genes after rhizobacteria inoculation to alleviate drought tolerance in sensitive rice genotype. Chem. Biol. Technol. Agric..

[51] Calvo P., Zebelo S., McNear D., Kloepper J., Fadamiro H. (2019). Plant growth-promoting rhizobacteria induce changes in *Arabidopsis thaliana* gene expression of nitrate and ammonium uptake genes. J. Plant Interact..

[52] Rennenberg H., Loreto F., Polle A., Brilli F., Fares S., Beniwal R.S., Gessler A.J.P.B. (2006). Physiological responses of forest trees to heat and drought. Plant Biol..

[53] Khan M.A., Asaf S., Khan A.L., Jan R., Kang S.M., Kim K.M., Lee I.J. (2020). Thermotolerance effect of plant growth-promoting Bacillus cereus SA1 on soybean during heat stress. BMC Microbiol..

[54] Mukhtar T., Rehman S.U., Smith D., Sultan T., Seleiman M.F., Alsadon A.A., Ali S., Chaudhary H.J., Solieman T.H., Ibrahim A.A., Saad M.A. (2020). Mitigation of heat stress in *Solanum lycopersicum* L. by ACC-deaminase and exopolysaccharide producing *Bacillus cereus*: effects on biochemical profiling. Sustainability.

[55] El-Daim A., Islam A., Bejai S., Meijer J. (2014). Improved heat stress tolerance of wheat seedlings by bacterial seed treatment. Plant Soil.

[56] Park Y.G., Mun B.G., Kang S.M., Hussain A., Shahzad R., Seo C.W., Kim A.Y., Lee S.U., Oh K.Y., Lee D.Y., Lee I.J. (2017). *Bacillus aryabhattai* SRB02 tolerates oxidative and nitrosative stress and promotes the growth of soybean by modulating the production of phytohormones. PLoS One.

[57] Misra S., Dixit V.K., Khan M.H., Mishra S.K., Dviwedi G., Yadav S., Lehri A., Chauhan P.S. (2017). Exploitation of agro-climatic environment for selection of 1-aminocyclopropane-1-carboxylic acid (ACC) deaminase producing salt tolerant indigenous plant growth promoting rhizobacteria. Microbiol. Res..

[58] Saleem A.R., Brunetti C., Khalid A., Della Rocca G., Raio A., Emiliani G., De Carlo A., Mahmood T., Centritto M. (2018). Drought response of *Mucuna pruriens* (L.) DC. inoculated with ACC deaminase and IAA producing rhizobacteria. PLoS One.

[59] Danish S., Zafar-ul-Hye M. (2019). Co-application of ACC-deaminase producing PGPR and timber-waste biochar improves pigments formation, growth and yield of wheat under drought stress. Sci. Rep..

[60] Pereira S.I.A., Abreu D., Moreira H., Vega A., Castro P.M.L. (2020). Plant growth-promoting rhizobacteria (PGPR) improve the growth and nutrient use efficiency in maize (*Zea mays* L.) under water deficit conditions. Heliyon.

[61] Zarei T., Moradi A., Kazemeini S.A., Akhgar A., Rahi A.A. (2020). The role of ACC deaminase producing bacteria in improving sweet corn (*Zea mays* L. var saccharata) productivity under limited availability of irrigation water. Sci. Rep..

[62] Niu X., Song L., Xiao Y., Ge W. (2018). Drought-tolerant plant growth-promoting rhizobacteria associated with foxtail millet in a semi-arid agroecosystem and their potential in alleviating drought stress. Front. Microbiol..

[63] Tiwari S., Lata C., Chauhan P.S., Nautiyal C.S. (2016). *Pseudomonas putida* attunes morphophysiological, biochemical and molecular responses in *Cicer arietinum* L. during drought stress and recovery. Plant Physiol. Biochem..

[64] Chandra D., Srivastava R., Gupta V.V., Franco C.M., Sharma A.K. (2019). Evaluation of ACC-deaminase-producing rhizobacteria to alleviate water-stress impacts in wheat (*Triticum aestivum* L.) plants. Can. J. Microbiol..

[65] Fasciglione G., Casanovas E.M., Quillehauquy V., Yommi A.K., Goñi M.G., Roura S.I., Barassi C.A. (2015). *Azospirillum* inoculation effects on growth, product quality and storage life of lettuce plants grown under salt stress. Sci. Hortic..

[66] Curá J.A., Franz D.R., Filosofía J.E., Balestrasse K.B., Burgueño L.E. (2017). Inoculation with *Azospirillum* sp. and *Herbaspirillum* sp. bacteria increases the tolerance of maize to drought stress. Microorganisms.

[67] Zhang M., Yang L., Hao R., Bai X., Wang Y., Yu X. (2020). Drought-tolerant plant growth-promoting rhizobacteria isolated from jujube (*Ziziphus jujuba*) and their potential to enhance drought tolerance. Plant Soil.

[68] Ortiz N., Armada E., Duque E., Roldán A., Azcón R. (2015). Contribution of arbuscular mycorrhizal fungi and/or bacteria to enhancing plant drought tolerance under natural soil conditions: effectiveness of autochthonous or allochthonous strains. J. Plant Physiol..

[69] Yasmin H., Rashid U., Hassan M.N., Nosheen A., Naz R., Ilyas N., Sajjad M., Azmat A., Alyemeni M.N. (2021). Volatile organic compounds produced by *Pseudomonas pseudoalcaligenes* alleviated drought stress by modulating defense system in maize (*Zea mays* L.). Physiol. Plantarum.

[70] Khan N., Bano A. (2019). Exopolysaccharide producing rhizobacteria and their impact on growth and drought tolerance of wheat grown under rainfed conditions. PLoS One.

[71] Gupta S., Pandey S. (2019). ACC deaminase producing bacteria with multifarious plant growth promoting traits alleviates salinity stress in French bean (*Phaseolus vulgaris*) plants. Front. Microbiol..

[72] Egamberdieva D., Wirth S.J., Shurigin V.V., Hashem A., Abd_Allah E.F. (2017). Endophytic bacteria improve plant growth, symbiotic performance of chickpea (*Cicer arietinum* L.) and induce suppression of root rot caused by *Fusarium solani* under salt stress. Front. Microbiol..

[73] Sarkar A., Ghosh P.K., Pramanik K., Mitra S., Soren T., Pandey S., Mondal M.H., Maiti T.K. (2018). A halotolerant *Enterobacter* sp. displaying ACC deaminase activity promotes rice seedling growth under salt stress. Res. Microbiol..

[74] Sapre S., Gontia-Mishra I., Tiwari S. (2018). *Klebsiella* sp. confers enhanced tolerance to salinity and plant growth promotion in oat seedlings (*Avena sativa*). Microbiol. Res..

[75] Kapoor R., Gupta M.K., Kumar N., Kanwar S.S. (2017). Analysis of nhaA gene from salt tolerant and plant growth promoting *Enterobacter ludwigii*. Rhizosphere.

[76] Patel S., Jinal H.N., Amaresan N. (2017). Isolation and characterization of drought resistance bacteria for plant growth promoting properties and their effect on chilli (*Capsicum annuum*) seedling under salt stress. Biocatal. Agric. Biotechnol..

[77] Nautiyal C.S., Srivastava S., Chauhan P.S., Seem K., Mishra A., Sopory S.K. (2013). Plant growth-promoting bacteria *Bacillus amyloliquefaciens* NBRISN13 modulates gene expression profile of leaf and rhizosphere community in rice during salt stress. Plant Physiol. Biochem..

[78] Shah G., Jan M., Afreen M., Anees M., Rehman S., Daud M.K., Malook I., Jamil M. (2017). Halophilic bacteria mediated phytoremediation of salt-affected soils cultivated with rice. J. Geochem. Explor..

[79] Li H., Lei P., Pang X., Li S., Xu H., Xu Z., Feng X. (2017). Enhanced tolerance to salt stress in canola (*Brassica napus* L.) seedlings inoculated with the halotolerant *Enterobacter cloacae* HSNJ4. Appl. Soil Ecol..

[80] Sapre S., Gontia-Mishra I., Tiwari S. (2022). Plant growth-promoting rhizobacteria ameliorates salinity stress in pea (*Pisum sativum*). J. Plant Growth Regul..

[81] Dixit V.K., Misra S., Mishra S.K., Tewari S.K., Joshi N., Chauhan P.S. (2020). Characterization of plant growth-promoting alkalotolerant *Alcaligenes* and *Bacillus* strains for mitigating the alkaline stress in *Zea mays*. Antonie Leeuwenhoek.

[82] Torbaghan M.E., Lakzian A., Astaraei A.R., Fotovat A., Besharati H. (2017). Salt and alkali stresses reduction in wheat by plant growth promoting haloalkaliphilic bacteria. J. Soil Sci. Plant Nutr..

[83] Damodaran T., Mishra V.K., Jha S.K., Pankaj U., Gupta G., Gopal R. (2019). Identification of rhizosphere bacterial diversity with promising salt tolerance, PGP traits and their exploitation for seed germination enhancement in sodic soil. Agric. Res..

[84] Hansen V., Bonnichsen L., Nunes I., Sexlinger K., Lopez S.R., van der Bom F.J.T., Nybroe O., Nicolaisen M.H., Jensen L.S. (2020). Seed inoculation with *Penicillium bilaiae* and *Bacillus simplex* affects the nutrient status of winter wheat. Biol. Fertil. Soils.

[85] Yousef N.M. (2018). Capability of plant growth-promoting rhizobacteria (PGPR) for producing indole acetic acid (IAA) under extreme conditions. Eur. J. Biol. Res..

[86] Chukwuneme C.F., Babalola O.O., Kutu F.R., Ojuederie O.B. (2020). Characterization of actinomycetes isolates for plant growth promoting traits andtheir effects on drought tolerance in maize. J. Plant Interact..

[87] Burt R. (2014).

[88] He Y., Pantigoso H.A., Wu Z., Vivanco J.M. (2019). Co-inoculation of Bacillus sp. and Pseudomonas putida at different development stages acts as a biostimulant to promote growth, yield and nutrient uptake of tomato. J. Appl. Microbiol..

[89] Mohanty P., Singh P.K., Chakraborty D., Mishra S., Pattnaik R. (2021). Insight into the role of PGPR in sustainable agriculture and environment. Front. Sustain. Food Syst..

[90] Kumar A., Patel J.S., Meena V.S., Srivastava R. (2019). Recent advances of PGPR based approaches for stress tolerance in plants for sustainable agriculture. Biocatal. Agric. Biotechnol..

[91] Khoshru B., Mitra D., Khoshmanzar E., Myo E.M., Uniyal N., Mahakur B., Das Mohapatra P.K., Panneerselvam P., Boutaj H., Alizadeh M., Cely M.V.T., Senapati A., Rani A. (2020). Current scenario and future prospects of plant growth promoting rhizobacteria: an economic valuable resource for the agricultural revival under stressful conditions. J. Plant Nutr..

[92] Singh H.B., Sarma B.K., Keswani C. (2016).

[93] Keswani C., Sarma B.K., Singh H.B., Singh H.B., Sarma B.K., Keswani C. (2016). Agriculturally Important Microorganisms: Commercial and Regulatory Requirement in Asia.

